# Perforated jejunal diverticulum as an unsual cause of acute abdomen: A case report

**DOI:** 10.1016/j.ijscr.2022.107130

**Published:** 2022-04-28

**Authors:** Atef Mejri, Khaoula Arfaoui, Mohamed Hedfi, Hakim Znaidi

**Affiliations:** a Department of General Surgery, Jendouba Hospital, Tunisia; b Department of General Surgery, Zaghouen Hospital, Tunisia; c Faculty of Medicine of Tunis, Tunis El Manar University, Tunis, Tunisia

**Keywords:** Emergency, Laparotomy, Peritonitis, Bowel resection, Jejunum

## Abstract

**Introduction and importance:**

Jejunal divertica is a rare entity with an often clinically silent course. However, it may be associated to life-threatening complications such as perforation. Therefore it should be considered in every case of acute abdomen.

**Case presentation:**

A 60-year-old female presented with a generalized abdominal pain associated with vomiting evolving for 24 h. Physical examination found an irreducible and tender hernia in the umbilical region with abdominal guarding. Laboratory test results showed a biological inflammatory syndrome. The primary diagnosis of strangulated umbilical hernia was suspected and the patient underwent an emergency laparotomy. Intra-operative examination revealed mutiple jejunal diverticula, with a perforation in one diverticulum leading to generalized peritonitis. A bowel resection and peritoneal lavage were performed with good outcome.

**Clinical discussion:**

Jejunal diverticula is a challenging condition with various non-specific clinical presentations. Jejunal perforation is its most feared complication. Deceitful abdominal examination among elderly patients and lack of specific signs may lead to diagnostic delay responsible for high mortality rate and poor prognosis. Adjunctive imaging modalities may be needed to help establish a prompt diagnosis and dictate management strategy. Treatment of perforated jejunal diverticulum is based on limited bowel resection associated to primary anastomosis.

**Conclusion:**

Jejunal diverticulitis should be kept in mind as a differential diagnosis in every case of acute abdomen. High index of clinical suspicion and eventual further radiological examinations are required to avoid misdaignosis and save patients' lives.

## Highlights

• Isolated jejunal diverticula perforation is extremely rare condition.
• Complicated jejunal diverticulitis is a diagnostic challenge with a high mortality rate.
• Jejunal diverticula might be difficult to identify and is rarely encountered by young surgeons.
• It is important to include this condition in the differential diagnosis of acute abdomen.

## 1 Introduction

Jejunal multiple diverticula represent an uncommon finding, reported in only 0.3 to 2.3% each year [1]. This acquired condition seems to result from intestinal dyskinesia and it usually remains silent. Nevertheless, it may lead to often undiagnosed signs of unexplained abdominal pain, intestinal malabsorption and occult gastrointestinal bleeding. However, it may result in acute more serious complications such as bowel obstruction, severe hemorrhage, diverticulitis, perforation and ensuing peritonitis with eventually fatal outcome [2]. Perfect awareness and early recognition of this entity is necessary to adapt management and reduce mortality rate.

Through this misleading featured case of a perforated jejunal diverticulum we aim to enlighten young unexperienced surgeons on the need to keep this condition into consideration in every case of acute abdomen.

This case report has been reported in line with the SCARE Criteria 2020 [3].

## 2 Presentation of case

**Fig. 1:**
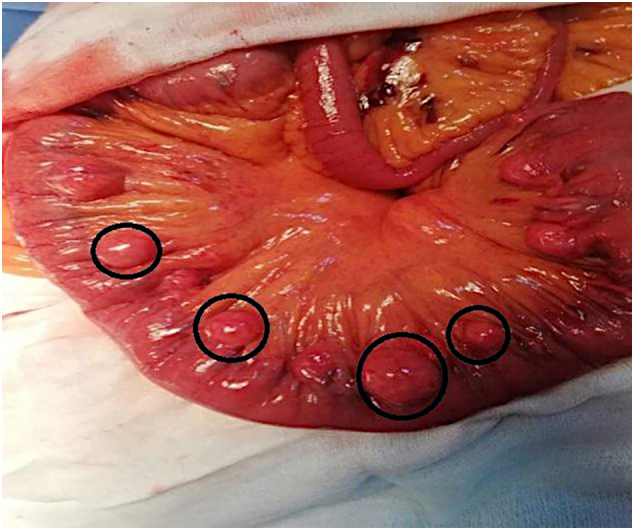
Intra-operative view showing multiple non complicated diverticula spread over the entire jejunum (black circles).

**Fig. 2:**
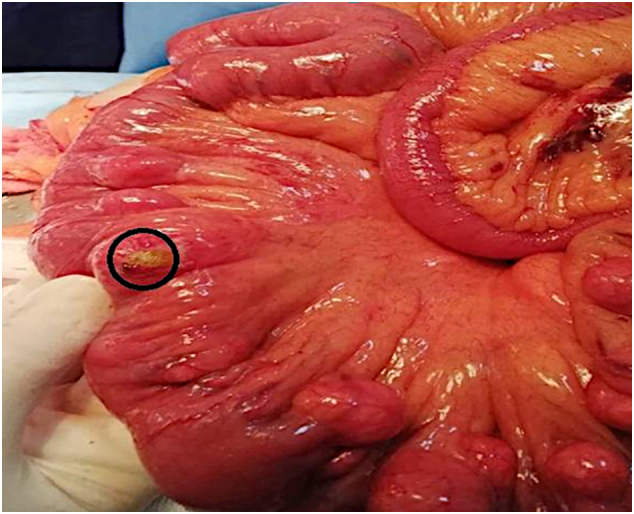
Intra-operative view showing the perforated jejunal diverticulum.

**Fig. 3:**
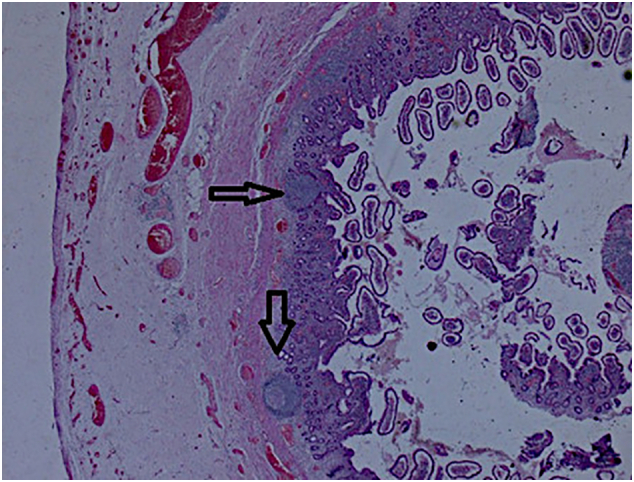
Pathological view showing nonspecific acute inflammatory changes with acute inflammatory infiltrate in the surrounding fat tissue (black arrows).

A 60-year-old female with no medical or surgical past history was admitted to the Department of General Surgery complaining of severe, generalized abdominal pain with vomiting. She had no significant past family history and she denied any tobacco, alcohol or any drug use. The pain suddenly started 24 h before admission and gradually increased. Physical examination revealed a blood pressure at 110/80 mmHg, a body temperature at 38.5 °C, a respiratory Rate at 22/min, and a heart rate at 106 beats/min. An irreducible and tender hernia was found in the umbilical region with abdominal guarding. Laboratory test results showed high levels of white blood cells (15,000 cells/Ul), high rate of C-reactive protein (100 mg/dL), and low levels of hemoglobin (10.6 g/dL). Whereas urea, creatinine, and lipase values were within the normal limits. The primary diagnosis of strangulated umbilical hernia was suspected and the patient underwent an emergency laparotomy, via median incision. The surgery was conducted by a senior surgeon with ten years surgical specialty experience. Afterward, a fibrinous exudate was noticed in the abdominal cavity. Exploration of the bowels (Fig. 1). One jejunal diverticulum, located at 40 cm distal to ligament of Treitz, was perforated (Fig. 2), and the omentum was gathered around the perforation in the epigastric area. The large intestines were found spared of any diverticula. A 40 cm small bowel resection taking away the perforated diverticulum was performed. A side-to-side jejuno-jejunal anastomosis was performed after peritoneal lavage. The postoperative course was uneventful. The surgical wound was clean. The patient was discharged on postoperative day 6. She was put on analgesics, oral antibiotherapy and venous thromboembolism prophylaxis. Medical follow-up visit on postoperative day twelve showed a fully-recovered and highly satisfied patient. The histopathological findings revealed nonspecific acute inflammatory changes with acute inflammatory infiltrate in the surrounding fat tissue (Fig. 3).

## 3 Discussion

Excluding Meckel's diverticulum, small bowel diverticula are acquired herniations of the mucosa and submucosa through weakened areas of the muscularis layer [2]. Being deprived of muscularis propria, these diverticula are atonic dilatations arising in the mesenteric border in contrast to Meckel's diverticulum which is located at the antimesenteric border [2]. They are commonly found on the mesenteric border where blood vessels pierce the muscularis layer [4]. Consequently, these diverticula might be difficult to identify when hidden in the mesenteric fat and they have a high risk of bleeding because they enter the bowel's blood supply site [5].

The most common part of the small bowel to be affected by diverticula is the proximal jejunum (75%), followed by the distal (20%) and then the ileum (5%) [4]. Furthermore, seventy seven percent of cases demonstrated multiple as opposed to solitary diverticula [6]. In spite the fact that the jejunum and the ileum are less frequently involved than the duodenum, diverticula in these two localisations are more prone to develop complications [4]. The increased incidence of diverticula in the proximal jejunum, compared to the distal jejunum and ileum, is attributed to the larger diameter of the blood vessels in the proximal jejunum, the vasa recta [7], [8]. Jejunal diverticulosis is a relatively rare condition with a reported annual incidence of 0.3–2.3% (1). Up to 60% of patients with jejunal diverticula may have concomitant colonic diverticula [9]. It is a disease of elderly people and is slightly more common in men (a male to female ratio of 2:1) [2], [8], [10]. Over 80% of affected individuals are in the 7th decade of life [11].

Jejunal diverticulum etiology has not been definitively identified. However, intestinal dysmotility which leads to abnormally elevated intraluminal pressure, and the structural weakness of penetration areas of the vasa recta blood vessels and nerves have been thought to play a role [1], [11], [12].

Most patients with jejunal diverticula are asymptomatic and in the majority of cases, jejunal diverticulosis is diagnosed incidentally either on imaging or intraoperatively [1]. Usually, this disorder is clinically silent until complicated [1], [10], [13]. Diverticulitis, which is the most common complication, may be asymptomatic or mimic the symptoms of irritable bowel syndrome: paroxysmal abdominal pain with cramping, dyspepsia, diarrhea, and occasional vomiting [8], [14]. More acute complications are perforation, massive hemorrhage, and intestinal obstruction, and are reported in approximately 10% of patients [4], [8].

Perforation of the jejunal diverticula is a serious complication that occurs in 2 to 6% of cases [14]. The clinical presentation of perforated jejunal diverticulitis is localized or diffuse peritonitis. However, perforation occurs primarily in the mesenteric leaves of the jejunum, resulting in a mesenteric abscess [4], which may lead to delayed diagnosis because conventional physical examination findings of an acute abdomen are absent. This delay in diagnosis can be disastrous in the case of frail or elderly patients [4]. Indeed, acute perforation is associated with a mortality rate of 40%, and delay in diagnosis or treatment can be fatal [7].

Diagnosis is challenging since there are no pathognomic signs or symptoms. Besides, elderly individuals may present a challenge in diagnosis due to the lack of clear physical signs, which explains the need for further examinations [10]. A plain abdominal radiography is the initial imaging modality of choice, since it can show signs of perforation such as the presence of free air, or signs of intestinal obstruction such as the presence of dilated intestinal loops and air-fluid levels [15]. Contrast enhanced CT is the most sensitive and most time-practical modality to identify such pathology in the critically unwell patient [16]. CT can visualize abnormalities associated with jejunal diverticulosis, including surrounding air in the mesentery, dilated small bowel loops with thickened walls, abscesses, inflammatory masses, and a hyperdense appearance of the mesentery [8], [13], [16]. Nevertheless, the best modality for identifying small bowel diverticula outside of a situation of emergency is MRI enteroclysis [16]. Furthermore, intra operatively, a diagnostic laparoscopy can be very useful [13].

According to the literature reporting 290 cases of jejunal diverticula [11], isolated jejunal diverticula perforation is extremely rare, and to date, only a few cases have been reported in the English literature [1]. Consequently, most centers have little experience in managing these cases. Since there is no grading system to stratify disease severity like clonic diverticulitis, many have adopted management strategies with a similar approach to colonic disease [1]. Asymptomatic jejunal diverticula do not call for any treatment. Non-operative treatment with bowel rest and antibiotics has been successful for cases of uncomplicated diverticulitis [1]. However, in case of complications, the recommended technique is resection of the involved intestinal segment with primary end to end anastomosis, in the absence of generalized peritonitis [14]. In case of panjejunoileal diverticulosis, resection may have to be limited to include only the segment containing the perforated diverticulum in order to avoid short bowel syndrome [2], [4], [14]. In all cases, we should keep in mind that perforated jejunal diverticula is associated to a high risk of mortality, ranging from 21 to 40%, which is closely related to the delay in diagnosis as well as the inherent risks associated with the old age of patients presenting with this disease [10].

## 4 Conclusion

Given its low incidence, complicated jejunal diverticulitis can be both a diagnostic and therapeutic challenge with a high mortality rate. Accordingly, although jejunal diverticulitis is rare, it is important to include it in the differential diagnosis of patients with abdominal pain. Indeed, early recognition and prompt surgical intervention are critical to improve patients' outcome.

## Abbreviations

CT: computed tomography
MRI: magnetic resonance imaging

## Funding

None.

## Ethical approval

An ethical approval was obtained from Jendouba Regional Hospital Medical Ethics Committee N° CC04Y22. We confirm that all methods were performed in accordance with the ethical guidelines of the 1975 Declaration of Helsinki.

## Consent

Written informed consent was obtained from the patient for publication of this case report and accompanying images. A copy of the written consent is available for review by the Editor-in-Chief of this journal on request.

## Authors' contributions

Conceptualization: AM.

Data curation: KA.

Supervision and performing surgery: HZ.

Writing - original draft:MH.

Writing - review & editing: AM.

The final version of manuscript was read and approved by all authors.

## Research registration

N/a.

## Guarantor

Atef Mejri.

## Provenance and peer review

Not commissioned, externally peer-reviewed.

## Declaration of competing interest

The authors declare that they have no conflicts of interest.

## References

[1] Leigh N., Sullivan B.J., Anteby R., Talbert S. (2020). Perforated jejunal diverticulitis: a rare but important differential in the acute abdomen. Surg. Case Rep..

[2] Luitel P., Shrestha B.M., Adhikari S., Kandel B.P., Lakhey P.J. (2021). Incidental finding of jejunal diverticula during laparotomy for suspected adhesive small bowel obstruction: a case report. Int. J. Surg. Case Rep..

[3] Agha R.A., Franchi T., Sohrabi C., Mathew G., Kerwan A., SCARE Group (2020). The SCARE 2020 guideline: updating consensus Surgical CAse REport (SCARE) guidelines. Int. J. Surg. Lond. Engl..

[4] Gupta S., Kumar N. (2017). Jejunal diverticula with perforation in non steroidal anti inflammatory drug user: a case report. Int. J. Surg. Case Rep..

[5] Ghandour R., Khalifeh G., Orm N.B., Rakka M., Dbouk S., El Sahili R., Mcheimeche H. (2020). Jejunal diverticular disease: a report of three cases. J. Surg. Case Rep..

[6] Saad M.K., Geahchan A., Ghandour F., Ghandour-Hajj F., Malouf H., Hajj I.E., Saikaly E. (2021). Midgut volvulus due to a true jejunal diverticula. Int. J. Rec. Surg. Med. Sci..

[7] Mantas D. (2011). Small intestine diverticula: is there anything new?. World J. Gastrointest. Surg..

[8] Martins B.A.Alves, Galletti R.Rodrigues, dos Santos Neto J.Marinho, Mendes C.Neiva (2018). A case of perforated jejunal diverticulum: an unexpected cause of pneumoperitoneum in a patient presenting with an acute abdomen. Am. J. Case Rep..

[9] Transue D.L., Hanna T.N., Shekhani H., Rohatgi S., Khosa F., Johnson J.-O. (2017). Small bowel diverticulitis: an imaging review of an uncommon entity. Emerg. Radiol..

[10] Ramzee A.F., Khalaf M.H., Ahmed K., Latif E., Aribi N., Bouchiba N., Singh R., Zarour A. (2020). Small intestinal diverticulosis: a rare cause of intestinal perforation revisited. Case Rep. Surg..

[11] Mansour M., Abboud Y., Bilal R., Seilin N., Alsuliman T., Mohamed F.K. (2022). Small bowel diverticula in elderly patients: a case report and review article. BMC Surg..

[12] Spasojevic M., Naesgaard J.M., Ignjatovic D. (2012). Perforated midgut diverticulitis: revisited. World J. Gastroenterol. WJG..

[13] Butler J.S., Collins C.G., McEntee G.P. (2010). Perforated jejunal diverticula: a case report. J. Med. Case Rep..

[14] Hadrich Z., Ben Ameur H., Masmoudi A., Zouari A., Boujelben S., Mzali R. (2021). Perforated jejunal diverticulitis with extensive diverticulosis: a case report. Clin. Case Rep..

[15] Gurala D., Idiculla P.S., Patibandla P., Philipose J., Krzyzak M., Mukherjee I. (2019). Perforated jejunal diverticulitis. Case Rep. Gastroenterol..

[16] Jambulingam R., Nanayakkara G. (2019). Non-operatively managed case of contained jejunal diverticular perforation. BMJ Case Rep..

